# Homeostatic dysregulation proceeds in parallel in multiple physiological systems

**DOI:** 10.1111/acel.12402

**Published:** 2015-09-29

**Authors:** Qing Li, Shengrui Wang, Emmanuel Milot, Patrick Bergeron, Luigi Ferrucci, Linda P. Fried, Alan A. Cohen

**Affiliations:** ^1^ Groupe de recherche PRIMUSDepartment of Family MedicineUniversity of Sherbrooke3001 12e Ave NSherbrookeQuebecCanadaJ1H 5N4; ^2^ Department of Computer ScienceUniversity of Sherbrooke2500 boulevard de l'UniversitéSherbrookeQuebecCanadaJ1K 2R1; ^3^ Translational Gerontology BranchLongitudinal Studies SectionNational Institute on AgingNational Institutes of HealthMedStar Harbor Hospital3001 S. Hanover StreetBaltimoreMaryland21225USA; ^4^ Mailman School of Public HealthColumbia University722 W. 168th StreetR1408New YorkNY10032USA; ^5^ Department of Chemistry, Biochemistry and PhysicsUniversité du Québec à Trois‐Rivières3351, boul. des ForgesC.P. 500Trois‐RivièresQCG9A 5H7; ^6^ Department of Biological SciencesBishop's University2600 Rue CollegeSherbrookeQCJ1M 0C8

**Keywords:** aging, biomarker, homeostasis, multi‐system dysregulation, physiology, statistical distance

## Abstract

An increasing number of aging researchers believes that multi‐system physiological dysregulation may be a key biological mechanism of aging, but evidence of this has been sparse. Here, we used biomarker data on nearly 33 000 individuals from four large datasets to test for the presence of multi‐system dysregulation. We grouped 37 biomarkers into six *a priori* groupings representing physiological systems (lipids, immune, oxygen transport, liver function, vitamins, and electrolytes), then calculated dysregulation scores for each system in each individual using statistical distance. Correlations among dysregulation levels across systems were generally weak but significant. Comparison of these results to dysregulation in arbitrary ‘systems’ generated by random grouping of biomarkers showed that *a priori* knowledge effectively distinguished the true systems in which dysregulation proceeds most independently. In other words, correlations among dysregulation levels were higher using arbitrary systems, indicating that only *a priori* systems identified distinct dysregulation processes. Additionally, dysregulation of most systems increased with age and significantly predicted multiple health outcomes including mortality, frailty, diabetes, heart disease, and number of chronic diseases. The six systems differed in how well their dysregulation scores predicted health outcomes and age. These findings present the first unequivocal demonstration of integrated multi‐system physiological dysregulation during aging, demonstrating that physiological dysregulation proceeds neither as a single global process nor as a completely independent process in different systems, but rather as a set of system‐specific processes likely linked through weak feedback effects. These processes – probably many more than the six measured here – are implicated in aging.

## Introduction

Research on aging biomarkers has traditionally focused on individual biomarkers; however, this has been changing as single‐mechanism explanations of aging such as oxidative stress, telomeres, and inflammation increasingly give way to multi‐factorial explanations, in which many mechanisms interact (Weinert & Timiras, 2003; Ferrucci, 2005; Fried *et al*., 2009; Cohen *et al*., 2013). In particular, much attention is focusing on physiological dysregulation (alternatively referred to as allostatic load or homeostenosis) (McEwen, 1998; Karlamangla *et al*., 2002; Crimmins *et al*., 2003). While evidence is abundant for increases in various types of physiological dysfunction with age, our use of ‘dysregulation’ is more restricted, as an emergent property of a complex system in the formal sense (Holland, 1992; Kauffman, 1993; Kriete, 2013). We define physiological dysregulation as the breakdown with age in the capacity of the complex regulatory networks to maintain organismal homeostasis due to changes in the state of these networks; we exclude from this definition adaptive changes with age and transient (i.e., reversible) responses to environmental challenges (Yashin *et al*., 2012).

This framework of homeostatic dysregulation supports the hypothesis that aging does not result from the downstream effects of a single factor, pathway, or process. Rather it suggests the following testable predictions: (i) multiple aging mechanisms should operate simultaneously; there could be several or many pathways, either independent or correlated (Kirkwood, 2005); (ii) markers of system state should be poorer predictors of aging‐related outcomes than measures of system dynamics (Varadhan *et al*., 2008; Yashin *et al*., 2010a); and (iii) risk of aging‐related outcomes (e.g., diseases) should often change as a function of deviations of parameters (e.g., biomarkers) from their normal ranges, rather than as a linear function of the parameters (Seplaki *et al*., 2005; Arbeev *et al*., 2011; Yashin *et al*., 2012; Cohen *et al*., 2013).

While these predictions are intuitive, they are hard to test and evidence has largely been lacking, in large part due to the difficulties in measuring dysregulation. Clearly, many studies have shown that physiological parameters change with age, and many of these changes are associated with specific pathologies or age‐related problems (e.g., Seplaki *et al*., 2005). In this sense, there has long been good evidence for functional changes during aging. However, theories on homeostatic dysregulation suggest that dysregulation is not a piece‐by‐piece set of problems that accumulate, but rather a breakdown in the functioning of a complex system such that the overall functional deficits cannot be simply or directly linked to specific piecewise problems. Perhaps the best evidence to date has come from two key studies: Lipsitz (2004) has demonstrated that declines in complexity of traits like heart rate variability are linked to aging, and Fried *et al*. (2009) showed coordinated, non‐linear changes with age in biomarkers representing several physiological systems.

Recently, we demonstrated a novel, rigorous way to measure dysregulation based on the statistical distance of a biomarker profile (Cohen *et al*., 2013, 2014; Milot *et al*., 2014b). Statistical distance assigns a score for how far an individual's profile is from the average profile; under a hypothesis of dysregulation, high scores indicate more dysregulation, and should thus increase with age and predict health outcomes after controlling for age. Furthermore, dysregulation is expected to be a higher order property of regulatory networks, and thus not overly sensitive to the choice of biomarkers (Cohen *et al*., 2013). These predictions have been confirmed in different human populations and species (Cohen *et al*., 2014; Milot *et al*., 2014a); however, it is not yet clear to what extent dysregulation might be a single global process, vs. a process that occurs independently or semi‐independently in different physiological systems which then further dysregulate each other. We might intuitively suspect that aging proceeds separately in different systems, but there are many known regulatory links across systems. For example, vitamin E plays roles in both the immune system and oxidative balance; many hormones coordinate activity across multiple systems; and relative levels of albumin and anemia appear to be coordinated (Cohen *et al*., 2012, 2015b). Additionally, the relative insensitivity of global dysregulation measures to biomarker composition gives the impression that dysregulation during aging might be best characterized as a single global process rather than a system‐by‐system process (Cohen *et al*., 2013, 2014). This paper investigates that question.

Here, we tested the relationships of dysregulation scores among different physiological systems using data on 37 common clinical blood biomarkers in four well‐known datasets, the National Health and Nutrition Examination Survey (NHANES), the Women's Health and Aging Study (WHAS), the Baltimore Longitudinal Study on Aging (BLSA), and *Invecchiare in Chianti* (Aging in Chianti, InCHIANTI). Biomarkers were grouped into six standard physiological systems, which we call the ‘*a priori* groupings’ (Table 1): immune function (five biomarkers), electrolytes (five biomarkers), vitamins (seven biomarkers), oxygen transport/anemia (eight biomarkers), lipids (four biomarkers), and liver/kidney/protein transport (eight biomarkers). We examined correlations among dysregulation levels of the *a priori* groupings to assess whether dysregulation rates are similar across physiological systems. To assess whether the *a priori* groupings optimally reflected the true underlying systems, we compared the correlations among them to correlations among randomly generated biomarker groupings (the ‘arbitrary groupings’), predicting lower correlations among dysregulation scores for groupings that better distinguished independent systems. If dysregulation proceeds completely independently across systems, correlations among dysregulation scores should be zero after age‐adjustment. Conversely, if there is a single, global dysregulatory process, dysregulation scores based on the *a priori* groupings should correlate as strongly among each other as based on the arbitrary groupings, and none of these correlations should be weak. We replicated our analyses across the four datasets and tested the associations of each system‐specific dysregulation with age, mortality, and various health outcomes.

**Table 1:** *A priori* biomarker groupings and summary statistics by dataset

Biomarker	System	Women's Health and Aging Study		InCHIANTI		Baltimore Longitudinal Study on Aging		National Health and Nutrition Examination Survey	
		Mean	SD	Mean	SD	Mean	SD	Mean	SD
Calcium	Electrolytes	9.5	0.5	9.4	0.5	9.3	0.4	9.5	0.39
Chloride	Electrolytes	103	4	106	4	104	3	103	2.84
Magnesium	Electrolytes	1.99	0.20	2.08	0.36	2.05	0.20	NA	NA
Sodium	Electrolytes	140.0	2.9	141.2	2.9	141.7	2.8	139	2.34
Potassium	Electrolytes	4.2	0.43	4.19	0.40	4.20	0.34	4	0.34
Phosphorous	Electrolytes	NA	NA	NA	NA	NA	NA	3.9	0.65
Hemoglobin	Blood measures	13.0	1.2	13.8	1.5	13.6	1.4	14	1.51
Hematocrit	Blood measures	39	4	41	4	41	4	41	4.41
Iron	Blood measures	80	27	85	29	89	32	86	36.5
Red cell distribution width	Blood measures	14.1	1.4	13.8	1.2	13.5	1.5	13	1.14
MCH	Blood measures	30.5	2.1	30.5	2.1	30.4	2.1	30	2.34
MCHC	Blood measures	33.1	1.2	33.7	1.0	33.5	1.2	34	0.91
Ferritin	Blood measures	112	124	123	127	107	99	81	118
Red blood cell count	Blood measures	4.26	0.43	4.53	0.47	4.50	0.48	4.7	0.48
Albumin	Proteins, liver, kidney	4.1	0.3	58.9	4.2	4.1	0.3	4.3	0.38
Alkaline Phosphatase	Proteins, liver, kidney	87	35	165	110	78	23	92	65
Total proteins	Proteins, liver, kidney	7.0	0.5	7.3	0.5	7.1	0.5	7.3	0.5
Gamma‐glutamyl transpeptidase	Proteins, liver, kidney	31	36	27	32	30	24	27	40.3
Lactate dehydrogenase	Proteins, liver, kidney	177	35	344	75	430	163	136	34.5
Uric acid	Proteins, liver, kidney	5.6	1.7	5.2	1.4	5.3	1.4	5.3	1.43
Alanine transaminase	Proteins, liver, kidney	19.6	10.9	20.8	10.5	32.0	12.4	24	24.1
Aspartate transaminase	Proteins, liver, kidney	16.2	12.1	19.4	15.2	28.1	10.6	25	17.8
White blood cell count	Immune measures	6.3	2.4	6.3	1.7	6.0	3.5	7.3	2.38
Neutrophil	Immune measures	60	10	59	9	55	10	55	11.9
Monocytes	Immune measures	6.9	2.4	6.6	2.2	9.2	4.3	8	2.39
Lymphocytes	Immune measures	29	9	31	8	32	10	33	10.7
Basophils	Immune measures	0.74	0.53	0.52	0.35	0.55	0.32	0.7	0.58
Triglycerides	Lipids	4.923	0.54	4.73	0.47	4.51	0.49	4.8	0.57
HDL	Lipids	3.968	0.29	57.1	15	58.7	17.2	53	16.2
Cholesterol	Lipids	224.3	41.3	215	41.8	194	36.5	200	42.9
LDL	Lipids	NA	NA	132	37.1	116	32.9	118	36.2
Cholesterol/HDL ratio	Lipids	4.471	1.46	NA	NA	NA	NA	NA	NA
Vitamin B12	Vitamins	494	307	471	334	NA	NA	641	2218
Folate	Vitamins	12.4	10.4	10.1	6.9	NA	NA	21	10.8
Vitamin A/retinol	Vitamins	72.06	23.6	1.94	0.49	NA	NA	52	17.6
Gamma‐tocopherol	Vitamins	10.07	1.13	2.21	0.95	NA	NA	224	121
Beta‐cryptoxanthin	Vitamins	0.148	0.14	0.21	0.16	NA	NA	11	8.29
Alpha‐carotene	Vitamins	0.104	0.1	0.06	0.05	NA	NA	3.7	5.33
Vitamin D‐25	Vitamins	21.71	10.9	54	36	NA	NA	22	8.88

## Results

### Dysregulation in *a priori* physiological systems

**Figure 1:**
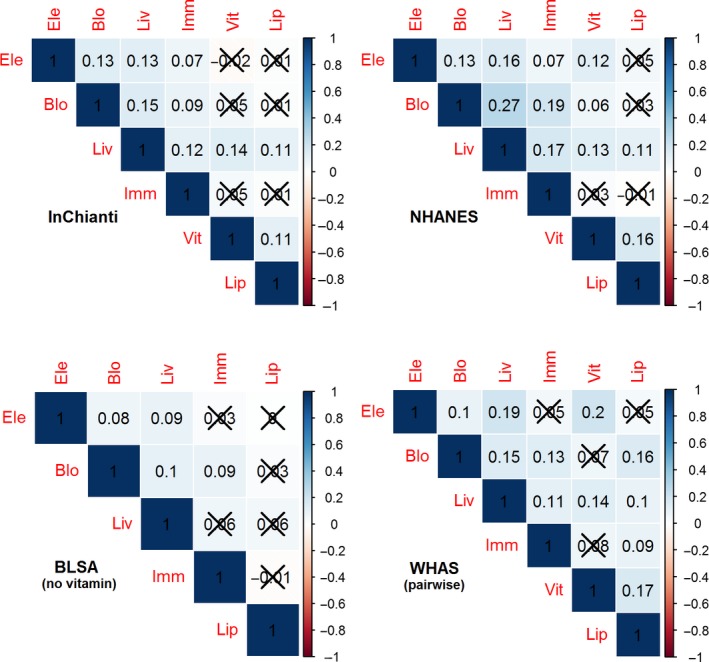
Correlations among age‐adjusted system‐specific dysregulation scores. The dysregulation scores were calculated from the six *a priori* biomarker groupings and then adjusted for age. Darker background color indicates stronger correlation, and values not significant at α = 0.05 are Xed out. The correlations are positive and weak in general, showing semi‐independence (or very weak dependence) of the six system‐specific dysregulation scores.

For each of the six physiological systems, we calculated a dysregulation score based on the Mahalanobis distance (see Experimental procedures) to measure dysregulation for each participant at each visit. Then we adjusted the system‐specific dysregulation scores for age and calculated the pairwise Pearson correlations among these residuals. This resulted in the 15 correlations in Fig. 1. We found weak but mostly significant correlations among dysregulation levels for the *a priori* groupings (Fig. 1). When significant, correlations were always positive, and never greater than *r = *0.27. Five correlations were significant in all data sets where they were tested, and two were significant in no data set (Fig. 1). The results in Fig. 1 are age‐adjusted (see Experimental procedures) but results without adjusting for age lead to the same conclusion (Fig. S1, Supporting Information).

### Correlations among *a priori* biomarker groupings compared with correlations among arbitrary biomarker groupings

If the *a priori* groupings accurately identify true physiological systems with respect to dysregulation, and if dysregulation proceeds at least semi‐independently in these systems, we expect weaker correlations among dysregulation scores of the *a priori* groupings compared to arbitrary groupings of biomarkers. This is because arbitrary groupings would mix biomarkers across physiological systems, and therefore make them less distinct and more correlated. Accordingly, testing the pairwise correlations among all possible combinations of biomarkers vs. the *a priori* groups allows us to assess not only whether there is separate dysregulation across systems, but whether our *a priori* groupings are representative, or whether there might be other hidden or non‐intuitive groupings of biomarkers that better represent the underlying dysregulation processes.

**Figure 2:**
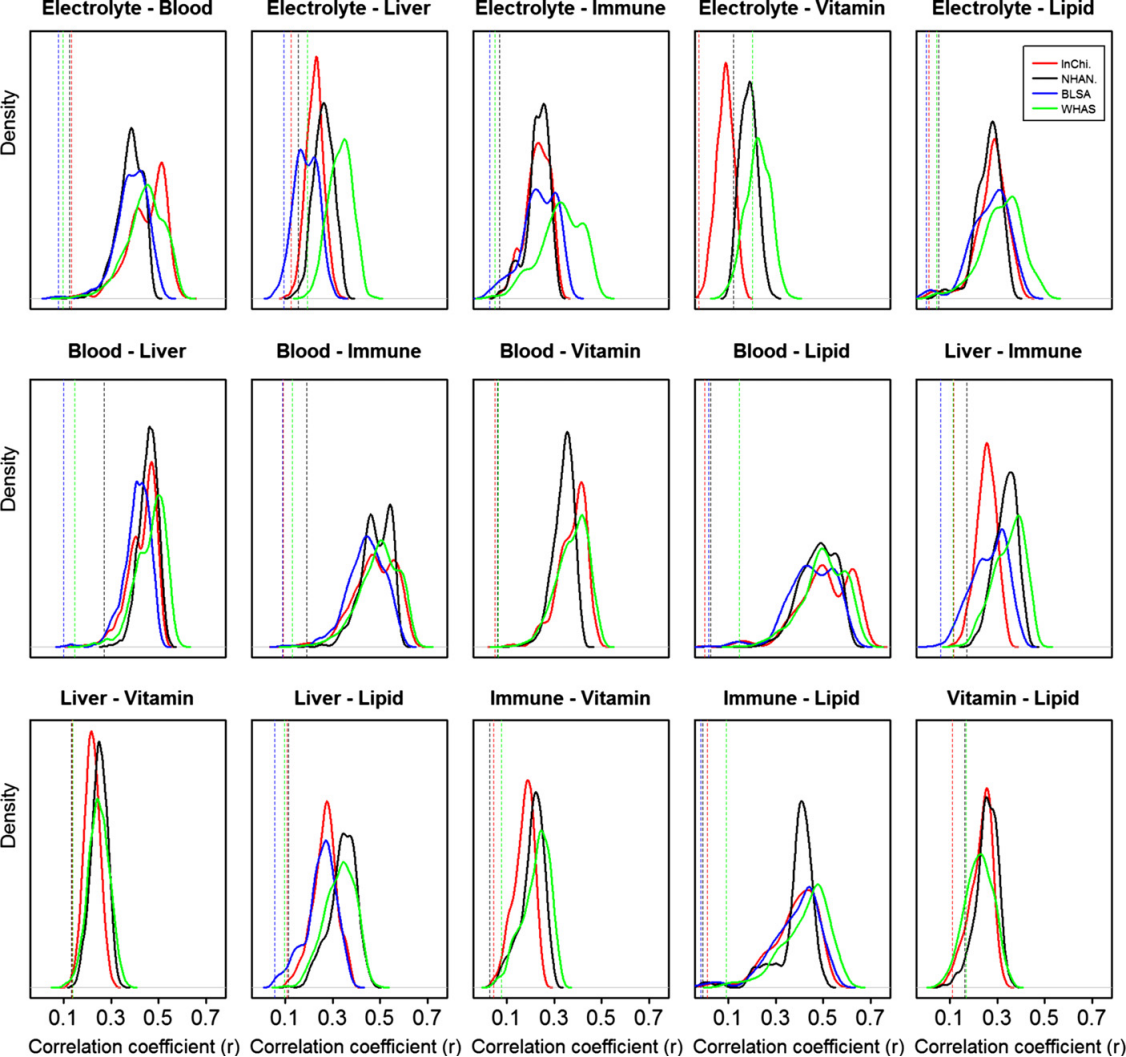
Quasi‐optimal separation of systems with *a priori* groups. The solid curves show the kernel densities by dataset of the correlation coefficients between two age‐adjusted dysregulation scores as calculated from all possible arbitrary biomarker groupings with the same sizes as the two *a priori* groups. Positions of the vertical dotted lines indicate correlations among the two age‐adjusted dysregulation scores corresponding to the *a priori* biomarker groupings, i.e., the results presented in Fig. 1. Each panel shows a possible pair of two systems. Different colors are used for different datasets. The figure shows that *a priori* biomarker groupings lead to much more weakly correlated dysregulation scores than arbitrary groupings and are close to as perfectly separated as possible, although a few correlations in the distribution are as low as the *a priori* correlations.

For each of the 15 pairwise combinations of the six physiological systems, we generated all possible biomarker groupings in two groups of equal size to the original two groups. For example, we had five electrolyte markers and four lipid markers, so for this pair we generated all possible combinations for two groups of five and four markers, respectively, 9 choose 5 = 9!/5!*(9–5)! = 126. However, we did not use groups with different sizes than the original (e.g., 6 and 3) to insure apples‐to‐apples comparisons. For each combination, we calculated the correlation between individuals’ age‐adjusted dysregulation scores as calculated for each biomarker group and compared this to the correlation between individuals’ dysregulation scores of the two original (*a priori*) biomarker groups (Fig. 2). To better visualize the results across the four datasets in a single panel, we show the kernel densities of the distributions of the correlations among the arbitrary groupings for each data set, and the vertical lines indicate the correlations for the *a priori* grouping. The vertical lines are almost always toward the extreme left of the distribution of correlations, indicating that the *a priori* groupings separated physiological systems about as well as possible. Note also that the kernel density distributions were generally concordant across datasets, but varied across the 15 pairs of systems.

However, our *a priori* groupings rarely produced the single most optimal division, i.e., the combination with the lowest correlation. Close examination of results revealed that this was because some markers have a strong affinity for one group or the other (e.g., the blood biomarkers hemoglobin, hematocrit, MCH, MCHC, red blood cell count, and RDW always group strongly), whereas other markers are not strongly associated with either group, and can thus be placed about equally well in either. Nonetheless, the performance of the optimal groups was never much better than the *a priori* groupings; for this reason, we believe that the *a priori* groupings are representative of their physiological systems and we used these *a priori* groupings in subsequent analyses.

### Association of dysregulation with age

**Figure 3:**
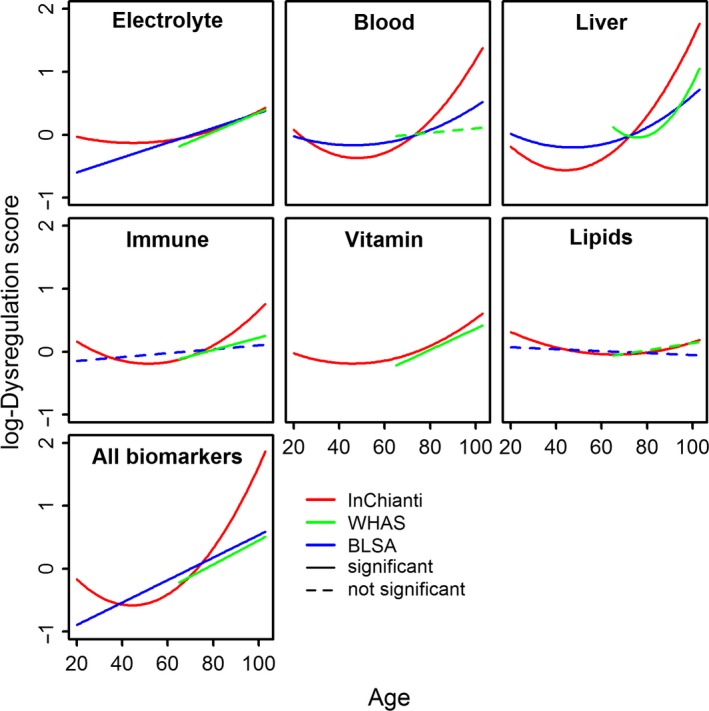
Changes in dysregulation scores with age, by physiological system. The first six panels show the association between age and dysregulation scores of the corresponding systems. The last panel shows the association between age and global dysregulation. We first fitted the quadratic model. If the quadratic term was significant (α = 0.05), we showed it with a solid quadratic curve. When the quadratic term was not significant, we fitted the linear model. Significant results are shown with a solid line and non‐significant results with a dashed line. Age started from 65 for Women's Health and Aging Study (WHAS) and the other two datasets had a small fraction of younger patients. The figure indicates a clear increase of system‐specific and global dysregulation scores with age. Note that the analyses here are longitudinal, so National Health and Nutrition Examination Survey (NHANES) data were not used.

Next, we calculated the association of system‐specific and global dysregulation with age for the three datasets that have longitudinal data, namely InCHIANTI, WHAS, and BLSA (Fig. 3). Dysregulation increased with age for most of the physiological systems in most data sets, with the exception of lipids in BLSA, where the slope was negative but not significant. All other results were positive although a few (dashed lines) were not significant. The quadratic term was always significant for InCHIANTI and sometimes significant for BLSA; the significant quadratic trajectories (J‐shaped) of dysregulation indicate an acceleration of dysregulation with age. In the same figure, we also show the result based on all of the 37 biomarkers (the last panel in Fig. 3), i.e., the association of a ‘global’ dysregulation with age; the results were always reproducible across the datasets and there do not appear to be marked differences in the rate of increase across systems, other than the potential absence of a relationship with age for lipid dysregulation.

### The effects of dysregulation on mortality and other health outcomes

**Figure 4:**
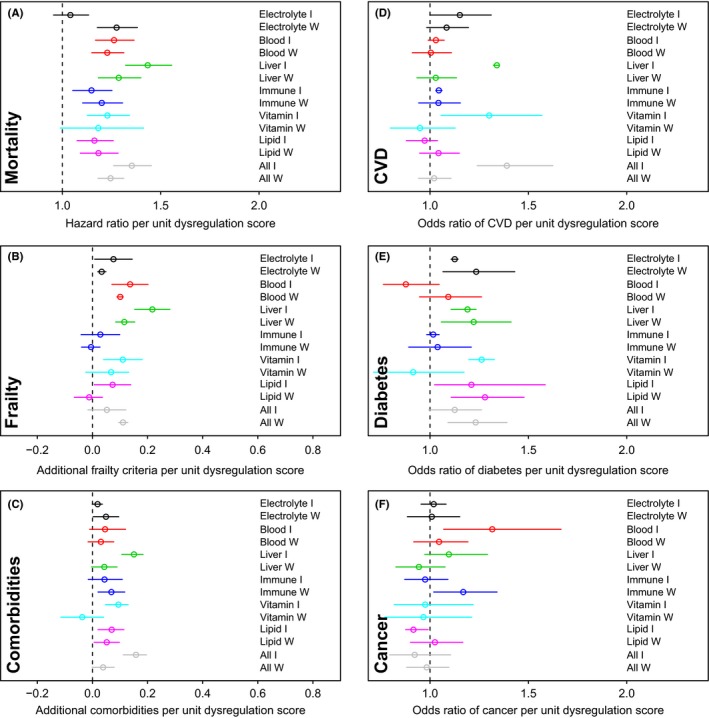
Relationships between dysregulation scores and health outcomes. Estimations (points) together with 95% CIs (segments) for relationships between health outcomes and dysregulation scores by physiological system, as well as global dysregulation scores. Results are based on regression models adjusting for age and sex. Different colors indicate different systems. ‘W’ indicates Women's Health and Aging Study (WHAS) and ‘I’ InCHIANTI. Associations between dysregulation scores and certain health outcomes are stronger, while the association is more ambiguous for CVD and not significant for cancer.

Finally, we explored the association of system‐specific and global dysregulation with mortality, clinical frailty, number of comorbidities, cardiovascular disease, cancer, and diabetes (Fig. 4) in the two datasets where the relevant information was available (InCHIANTI and WHAS), as done previously for global dysregulation on a subset of markers (Milot *et al*., 2014b). Depending on data availability, we examined either cross‐sectional associations or longitudinal associations; see Experimental procedures for details.

Dysregulation predicted mortality controlling for age, usually significantly and quite strongly (Fig. 4a), with each additional unit of dysregulation score implying about a 30% increase in hazard of mortality. The sole exception was electrolyte dysregulation in InCHIANTI; blood, liver, and global dysregulation showed the strongest effects. Frailty was predicted by electrolyte, blood, and liver dysregulation, and perhaps weakly by vitamin, lipid, and global dysregulation (Fig. 4b). Dysregulation was only weakly associated with the number of comorbidities, but effects were surprisingly stable across systems and data sets (Fig. 4c). Cardiovascular disease incidence is strongly predicted by liver, vitamin and global dysregulation, and more weakly by electrolyte and/or blood dysregulation in InCHIANTI, but the results do not hold for cross‐sectional analysis of CVD prevalence in WHAS (Fig. 4d). Diabetes incidence and prevalence are reliably predicted by electrolyte, liver, lipid, and global dysregulation (Fig. 4e). Cancer was generally unassociated with dysregulation, with an exception for cancer incidence being predicted by blood dysregulation in InCHIANTI (Fig. 4f).

### Feedback effects among systems

We tested the potential for long‐term causal effects among the six systems, using structural equations models to assess the effect of dysregulation in each system on all the others at subsequent time points and controlling for the effect of each dysregulation on itself. Each system's dysregulation consistently predicted itself at subsequent time points (*P* < 0.0001 for all systems in all data sets), but in no case was there a clear, reproducible result for dysregulation scores of one system predicting another in more than two datasets (Table 2). Detailed results are available in Table S2.

**Table 2:** Significant temporal predictions of inter‐system dysregulation scores identified using structural equations models

Baltimore longitudinal study on aging (5 systems)	Women's Health and Aging Study (5 systems)	InChianti (5 systems)	InChianti (6 systems)
Blood → Electrolyte	Lipid → Blood	Lipid → Electrolyte	Lipid → Electrolyte
Electrolyte → Lipid	Electrolyte → Liver	Immune → Blood	Immune → Blood
	Liver → Electrolyte	Liver → Electrolyte	Blood → Liver
	Electrolyte → Immune		Vitamin → Immune
			Liver → Vitamin

The signs of the two arrows ‘Lipid → Electrolyte’ and ‘Immune → Blood’ are negative. Relationships listed are those significant at α = 0.05, among the 20 tested in datasets with five systems and 30 tested in datasets with six systems. Note that no relationship was replicated in more than two datasets, and only three (Liver → Electrolyte, Lipid → Electrolyte, and Immune → Blood) were replicated in two systems.

## Discussion

Overall, we found strong support for the existence of system‐specific dysregulation processes in all six physiological systems we tested. We also found semi‐independence between the six system‐specific dysregulation processes. *A priori* definition of these systems by biomarker groupings was close to optimal within the marker set available, though some biomarkers did not clearly fall within any system. Dysregulation of all systems except lipids clearly increased with age, and in some cases clearly accelerated with age. System‐specific dysregulation scores also predicted a wide variety of health outcomes, though these associations often depended on which system, which outcome, and whether the association was analyzed for cross‐sectional prevalence or longitudinal incidence. Mortality and frailty in particular were predicted independently by dysregulation of most systems. Correlations among individuals’ dysregulation scores for different systems were mostly positive but weak, suggesting a model of semi‐independence, i.e., that processes internal to each system cause dysregulation, but with the possibility for feedback effects with dysregulation of other systems.

This finding has substantial implications for our understanding of the biological mechanisms of aging. For example, if aging is largely a result of uniform cellular senescence, we would not expect largely independent dysregulation processes in different systems. More likely, cellular senescence interacts with tissue‐, organ‐, and organism‐level processes in complex, system‐specific ways. In this case, a focus on cellular senescence alone as the root of aging may be misplaced. Conversely, while global dysregulation can be measured, it does not appear that there is a single, organism‐level dysregulation process that can explain aging. Our findings thus support the need to incorporate multiple hierarchical levels to arrive at a comprehensive understanding of aging.

These findings also present the first unequivocal demonstration of integrated multi‐system physiological dysregulation during aging. While numerous authors have discussed the possibility of multi‐system dysregulation (Crimmins *et al*., 2003; Ferrucci, 2005; Seplaki *et al*., 2005; Varadhan *et al*., 2008; Fried *et al*., 2009; Arbeev *et al*., 2011; Maggio *et al*., 2014), empirical studies have been sparse. Lipsitz has demonstrated loss of complexity in cardiac rhythms and other aspects of physiology (Lipsitz, 2004), but the link to dysregulation is still unclear. Allostatic load studies have quantified what can be interpreted as a global measure of dysregulation (Karlamangla *et al*., 2002; Crimmins *et al*., 2003; Szanton *et al*., 2009), comparable to some of our previous studies using statistical distance. To our knowledge, the only study to explicitly measure multi‐system physiological dysregulation is that of Fried *et al*. (2009). In that study, one or two biomarkers per system were used to define dysregulation based on *a priori* clinical knowledge; number of dysregulated systems was then shown to predict clinical frailty status. Our findings here present a substantial next step: (i) using a previously validated statistical method to quantitate dysregulation; (ii) including 4+ markers per system to increase the robustness of the inference; (iii) independently validating the choice of biomarkers in each system; (iv) establishing the correlations among dysregulations of different systems; (v) using longitudinal data to make more robust temporal inferences; and (vi) explicitly testing the relationship of each type of dysregulation with age, mortality, and chronic diseases.

The most likely explanation for the weak correlations among dysregulation scores of different systems is that there are feedback effects among the systems. We attempted to test for this using the structural equation models to predict subsequent dysregulation scores based on current dysregulation scores across systems. While some results were significant, this proportion was only slightly higher than might be expected by chance given multiple testing issues (Rothman, 1990; Bender & Lange, 2001), and the relationships we did find were not easily reproducible across datasets. We see three likely explanations for this failure to detect clear feedback effects. First, the six systems measured here are a very small percentage of the actual systems involved in dysregulation and aging; they are the ones for which sufficient biomarker data were available in our datasets. It is thus highly likely that any feedback effects among these six systems are mediated by other systems that have not been assessed. This would explain why we do find some significant effects, but inconsistently so across datasets: contingency could have a large role. Second, timescales could be crucial here, and perhaps the intervals of several years between the visits (somewhat variable across datasets) were not the right timescale to detect the effects we were looking for. Third, there could be some upstream process causing all the dysregulations, such that they are correlated but do not cause each other.

Obviously, the 37 biomarkers measured here represent a tiny fraction of the molecules and systems likely to be implicated in aging (Medvedev, 1990; Kirkwood, 2011). Similar methods can likely be applied to high‐throughput technologies such as microarray, proteome, and metabolome data to identify other important systems (e.g., Hoffman *et al*., 2014). Nonetheless, circulating blood biomarkers should provide much of the most important information: organism‐level signaling occurs mostly through the circulatory and central nervous systems, and many of the classical biomarkers are critical regulatory molecules with broad roles, or are well‐known to be good general indicators of health state. Moreover, our previous studies show that the signal of dysregulation appears to get stronger as more markers are included, but with diminishing returns for additional markers (Cohen *et al*., 2013, 2014). At this point, we do not feel there is sufficient evidence to thoroughly explore the implications of our findings system‐by‐system. However, long term our hope is that the approach we describe can be used to gradually work down from the organism level to physiological systems, organs, tissues, and cells. This is another advantage of our approach relative to other methods of measuring allostatic load or organism state: we provide a pathway toward linking an organismal understanding to more detailed mechanistic studies.

The presence of global and system‐specific dysregulation, as measured by statistical distance, supports a complex systems theory of aging in which individual molecules play minor roles in determining overall system state and behavior (Ferrucci, 2005; Managbanag *et al*., 2008; Fried *et al*., 2009; Cohen *et al*., 2013). Under this theory, aging is at least partly due to emergent properties of complex system dynamics. This is further strengthened by the robustness of dysregulation measures to the inclusion or exclusion of individual biomarkers. Correlations of individual biomarkers with age vary substantially across populations and must be interpreted with caution (Cohen *et al*., 2015b); likewise, the same biomarker can have both pro‐ and anti‐longevity associations at different points in the lifespan (Moeller *et al*., 2014). These findings might arise because aging involves a mix of non‐adaptive (i.e., pathological) changes, as well as other compensations to these changes. This is supported by findings that optimal biomarker levels change with age (Arbeev *et al*., 2011). Accordingly, while our results agree with a complex systems theory of aging, they are not proof of such an explanation. In particular, we cannot exclude the possibility of an upstream cause that affects dysregulation in multiple systems.

Likewise, a complex systems understanding of aging and physiology suggests that targeting individual molecules as key players in aging and chronic disease will rarely bear fruit; in this context, measures of system‐specific dysregulation will simultaneously provide a big‐picture explanation of the physiological underpinnings of aging pathologies and concrete tools to measure aspects of biological aging rate (Levine, 2013). For example, we can ask how aspects of lifestyle such as diet, physical activity, and social participation affect dysregulation rates in different systems, targeting the most critical (such as blood and liver dysregulation here) for interventions. Quantitation of system‐specific dysregulation thus provides both biological insight into the aging process and concrete tools to measure aging and improve the health of aging populations.

Our study must be considered in light of several limitations. First, statistical distance depends on identifying a ‘normal’ physiological state, the statistical centroid. This is usually calculated as the population average for all parameters, a reasonable but imperfect approximation. It is not easy to find an ideal centroid (Cohen *et al*., 2013, 2015a); hence the dysregulation scores could be biased systematically. Accordingly, further work is needed to estimate a robust vector of biomarker values to replace the centroid, based not on the means but, ideally, on age‐specific profiles of mortality risk across biomarker values. Second, the suite of biomarkers used is neither comprehensive nor the best conceivable. The statistical methods used are designed to function even with imperfect biomarkers, but undoubtedly future studies will be necessary to improve biomarker selection and thereby produce more accurate results. Third, we do not take genetic background into account. While genetic factors certainly have some influence on the processes we seek to describe, genetic control of aging appears largely due to many genes of small effect (Yashin *et al*., 2010b), many of which have yet to be identified, and incorporation into this study would require stratification into more groups than is feasible. Nevertheless, we believe that both genetic and sociological influences on aging pass through the physiological pathways studied here, making them logical follow‐up studies when more information is available.

## Experimental procedures

### Datasets

Sampling and data collection procedures for the four study populations are described in detail elsewhere (Shock, 1984; Fried *et al*., 1995; Guralnik *et al*., 1995; Ferrucci *et al*., 2000; Ferrucci, 2009); we also provide more detailed information in the Supporting Information. BLSA, InCHIANTI, and WHAS are longitudinal cohort studies and are composed of elderly community dwelling adults (BLSA and InCHIANTI contain a small proportion of young individuals). The sample sizes used for this study were 2644 visits (1256 patients), 2932 visits (1308 patients), and 3799 visits (1226 patients) for BLSA, InCHIANTI and WHAS, respectively. WHAS combines the WHAS I and WHAS II studies, and is solely women aged 65+. NHANES is a cross‐sectional study based on a representative sample of the US population and conducted in various waves since the 1970s; we combine data from six waves (1998–2007) which yielded a sample of 29 188 patients. The analysis of semi‐independence of different aging systems was performed on all of the four datasets. The effect of age on dysregulation was performed on the three longitudinal datasets. The analysis of health outcomes were performed on InCHIANTI and WHAS.

### Biomarker selection

Biomarkers were chosen based on availability in sufficient sample size across the four studies. In a few cases, biomarker groupings differed slightly across datasets due to data availability, but we assured the same number of biomarkers per group in each dataset (Table 1). Due to missing data, we did not include vitamins in the analyses of BLSA. Folate, total vitamin D‐25 and vitamin B12 were only available at baseline in InCHIANTI, so we included these biomarkers in the cross‐sectional analysis of biomarker correlations between biomarker groups (Figs 1 and 2), but did not include them in the longitudinal analyses showing associations between age and dysregulation scores and associations between dysregulation scores and health outcomes (Figs 3 and 4). The biomarkers are listed in Table 1 with their means and standard deviations, organized by *a priori* physiological system. The need for a largely common list of biomarkers across four datasets resulted in a final list that was composed nearly exclusively of markers that are (i) common; (ii) used often in clinic; and (iii) cheap, increasing the relevance of any results for clinical implementation. Assignment of markers to *a priori* systems was based on consultation among biologists on the team.

### Dysregulation scores

Recently, we proposed a novel way to measure physiological dysregulation based on clinical biomarkers (Cohen *et al*., 2013) and we use this method to measure dysregulation in this paper. Under the hypothesis that a well‐functioning, homeostatic physiology should be relatively similar across individuals, but that there are many ways in which physiology might become dysregulated, we proposed statistical distance, specifically Mahalanobis distance, (Mahalanobis, 1936) as a measure of physiological dysregulation. The Mahalanobis distance applied to biomarkers is a measure of how aberrant an individual's profile is relative to everyone else in the population, and greater distance should thus measure greater dysregulation. The Mahalanobis distance also has certain advantages over other multivariate distances. For example, the Euclidean distance is a special case of the Mahalanobis distance when variables are uncorrelated with each other (Tan *et al*., 2006); by taking into account correlation structure, Mahalanobis distance automatically corrects for redundancy among variables.

Normally, statistical distance is calculated based on the entire population, but this is not optimal when it is interpreted as physiological dysregulation, because the ‘normal’ state is defined as the centroid of the entire population. Using a younger, healthier reference population to calculate the centroid provides a better signal, though choice of reference population is not critical unless it differs substantially from the study population in multiple demographic characteristics (Cohen *et al*., 2015a). Here, we used the younger patients as the reference group, and calculated the Mahalanobis distance from each patient to the centroid of the reference group in the multivariate biomarker space. Age structure is different in the four datasets, so we took different age thresholds for different datasets based on the principle that the reference group should consist of relatively younger patients in each dataset and sample size of the reference group should not be too small. For NHANES we took patients under 45 years old as reference group, 55 for BLSA, 65 for InCHIANTI and 75 for WHAS. In each case we used the biomarker values from the first visit of each eligible patient to make the reference group. To confirm that results were not sensitive to choices of age thresholds, we reran each model using two additional manually chosen thresholds within 5 years of those listed above, but found no qualitative differences in results (data not shown). This is expected based on our previous validation study (Cohen *et al*., 2015a). The system‐specific dysregulation scores were compared with global dysregulation scores based on all biomarkers.

### Data analysis

Changes in dysregulation with age were studied using Bayesian linear mixed models with uninformative priors and an individual intercept. We first fitted the quadratic model; if the quadratic term was not significant we fitted the linear model; and if the linear model was still not significant we show a dashed line in the figure. Age started from 65 for WHAS (green lines) whereas the other two datasets had some younger patients, though they were still primarily composed of individuals aged 65+. All analyses were performed in r v3.0.1. All codes are available upon request.

### Data transformation

All biomarkers were transformed before analysis. The variables were log‐ or square‐root‐ transformed as necessary to approach normality. All variables were centered at the mean of the reference group and divided by the standard deviation of the reference group.

### Correlation between dysregulation scores of *a priori* systems

Previous studies (Cohen *et al*., 2013, 2014; Milot *et al*., 2014b) showed that global dysregulation increased with age, and the current study confirms that this is also true for system‐specific dysregulation (see ‘association of dysregulation with age’). Accordingly, correlations among dysregulation scores of different systems might be due solely to the fact that each correlates with age, rather than to an independent biological link in the dysregulation rates. We thus adjusted for age before measuring correlations between system‐specific dysregulation scores. We did this by calculating the residuals (predicted values) of the dysregulation scores after running a locally weighted regression in each case (R function loess()), since the change of dysregulation scores with age is likely to be non‐linear and we wanted to capture the full relationship as faithfully as possible. We also replicated the analysis without adjusting for the effect of age (Fig. S1).

For NHANES, we had only one visit per patient. For cross‐sectional analyses of the three longitudinal datasets, we randomly selected a visit with complete data for each patient to eliminate intra‐patient correlations. For InCHIANTI, this corresponded exactly to the first visit because several biomarkers were not taken during the follow‐up visits. We calculated the Pearson correlations among the age‐adjusted dysregulation scores as reported in Fig. 1. For example, the *a priori* system of electrolytes contained five biomarkers and the *a priori* system of vitamins consisted of seven biomarkers; we obtained the age‐adjusted dysregulation scores for the electrolyte system based on the five biomarkers and the age‐adjusted dysregulation scores for the vitamin system based on the seven biomarkers. In this way, each patient had two new variables, one representing dysregulation in the electrolyte system and the other representing dysregulation in the vitamin system, and we calculated the correlation between the two variables to measure the dependence level between the two system‐specific dysregulation processes. This resulted in the ‘Electrolyte‐Vitamin’ correlation in Fig. 1.

As information on vitamins was largely unavailable for BLSA, we decided not to include vitamin dysregulation for BLSA. For WHAS, there was no patient with complete information on all 37 biomarkers, so we reported pairwise correlations on available observations.

### Correlations among all possible biomarker groupings

The same biomarkers were used to calculate correlations among age‐adjusted dysregulation scores of all possible combinatorial biomarker groupings with equal size to the *a priori* biomarker groupings. There are $\frac{6\times (6-1)}{2}$ = 15 correlations among the six *a priori* biomarker groups. For each of the 15 cases, we took all biomarkers in the two *a priori* groups, divided them into two random biomarker groups with equal size to the *a priori* groups, and calculated the correlation between the dysregulation scores of the two random biomarker groups (representing system‐specific dysregulation in two ‘random’ physiological systems). We then repeated this for all combinatorial divisions of the biomarkers into sets of appropriate size. Taking the same example of electrolytes and vitamins, there are 12 biomarkers altogether from the two *a priori* biomarker groups. Since the two *a priori* groups had five and seven biomarkers respectively, we analyzed always of dividing the 12 biomarkers into two groupings, one with five biomarkers and the other with seven. As a result, there were $C_{12}^{5}$ = 792 possibilities to make two arbitrary biomarkers groupings having equal size with the two original groups. In each of the 792 cases, we calculated age‐adjusted dysregulation scores based on the two arbitrary biomarker groups, and then calculated the correlation between these two age‐adjusted dysregulation scores. This generated 792 correlation coefficients. We showed the four kernel density distributions for the four datasets of the 792 correlations in the panel ‘Electrolyte‐Vitamin’ in Fig. 2; to compare with the *a priori* biomarker grouping, we also showed the correlation between the *a priori* dysregulation scores with vertical dotted lines; positions of the vertical dotted lines on the *x*‐axis indicate the correlations, i.e., the values presented in Fig. 1.

### Association of dysregulation scores with age

Trajectories of dysregulation scores with age were estimated for each physiological system, and also for the global dysregulation score calculated with all of the 37 biomarkers, for the three datasets with longitudinal information. We used Bayesian mixed models implemented in R (package mcmcglmm) with uninformative priors, 17 000 iterations and burn‐in at 7000, which was always sufficient to insure auto‐correlations in Markov chain samples of < 0.1. Models included population‐ and individual‐level intercept, age, and square of age at each visit. Inclusion or exclusion of individual‐level terms had little effect on fixed effect estimates.

### Association of health status with dysregulation scores

For InCHIANTI and WHAS, for which we had access to health status information, we ran a series of regression models to predict health status based on dysregulation scores controlling for age (and for sex in InCHIANTI). Due to data availability, frailty was analyzed longitudinally in WHAS and cross‐sectionally in InCHIANTI, while chronic diseases were analyzed longitudinally in InCHIANTI and cross‐sectionally in WHAS. The relationship between dysregulation scores and mortality was assessed using time‐to‐event Cox proportional hazards models with age as the timescale. Frailty criteria and number of comorbidities were assessed using Poisson regression. Logistic regression was used for individual chronic diseases. Age was rigorously controlled for using a flexible cubic basis spline (bs function, fda package, r) with four knots. The Poisson regressions and logistic regressions were implemented with the MCMC Bayesian generalized linear mixed model (mcmcglmm package), when longitudinal data were available, controlling for individual as a random effect. When the data were cross‐sectional, we used the glm() function. We tested the relationship between each system's dysregulation and each health outcome both with and without control for dysregulation of all other systems.

There are many other determinants of health status that we do not control for, nonetheless, the notion of causality in complex dynamic systems does not apply in the same way it does to simple, deterministic systems (Wagner, 1999); accordingly, we were not attempting to show even putative causal relationships between dysregulation scores and health outcomes, but rather consistent and strong associations indicating a close linkage of the phenomena within network dynamics.

### Structural equations models

To assess possible feedback effects among dysregulations in different systems, we employed Seemingly Unrelated Regression (SUR; Zellner, 1962), a variant on structural equations models. These models permitted us to test for the effects of each system on each other system, controlling for the effects of all the other systems. Models used lagged effects such that we were always assessing the effect of a dysregulation level at a given visit on the level of another dysregulation level at the subsequent visit. We were not able to use this longitudinal approach to the vitamin system except for InCHIANTI.

## Funding info

AAC is a member of the FRQ‐S‐supported *Centre de recherche sur le vieillissement* and *Centre de recherche du CHUS*, and is a funded Research Scholar of the FRQ‐S. This research was supported by CIHR grant #s 110789, 120305, 119485 and by NSERC Discovery Grant # 402079‐2011, as well as by the Intramural Research Program of the National Institute on Aging.

## Conflict of interest

None declared.

## Author contributions

QL and AAC conceived the study, carried out analyses, and wrote the manuscript. SW, EM, and PB consulted on analyses. LF and LPF provided data. All authors contributed to interpretation and provided comments on the manuscript.

## Supporting information


**Fig. S1** Correlations among dysregulation scores of the a priori systems. The only difference from Fig. 1 in the main text is we did not adjust for age.

**Table S1** Results from structural equations models showing lagged effects of dysregulation across systems.
**Appendix S1** Additional details on data sets, health outcomes measures, and structural equations models.
