# Supplementary figures and images for: Homeostatic dysregulation proceeds in parallel in multiple physiological systems
Source: Aging Cell. 2015 Sep 29;14(6):1103–12. doi: 10.1111/acel.12402 (PMC4693454; doi:10.1111/acel.12402)

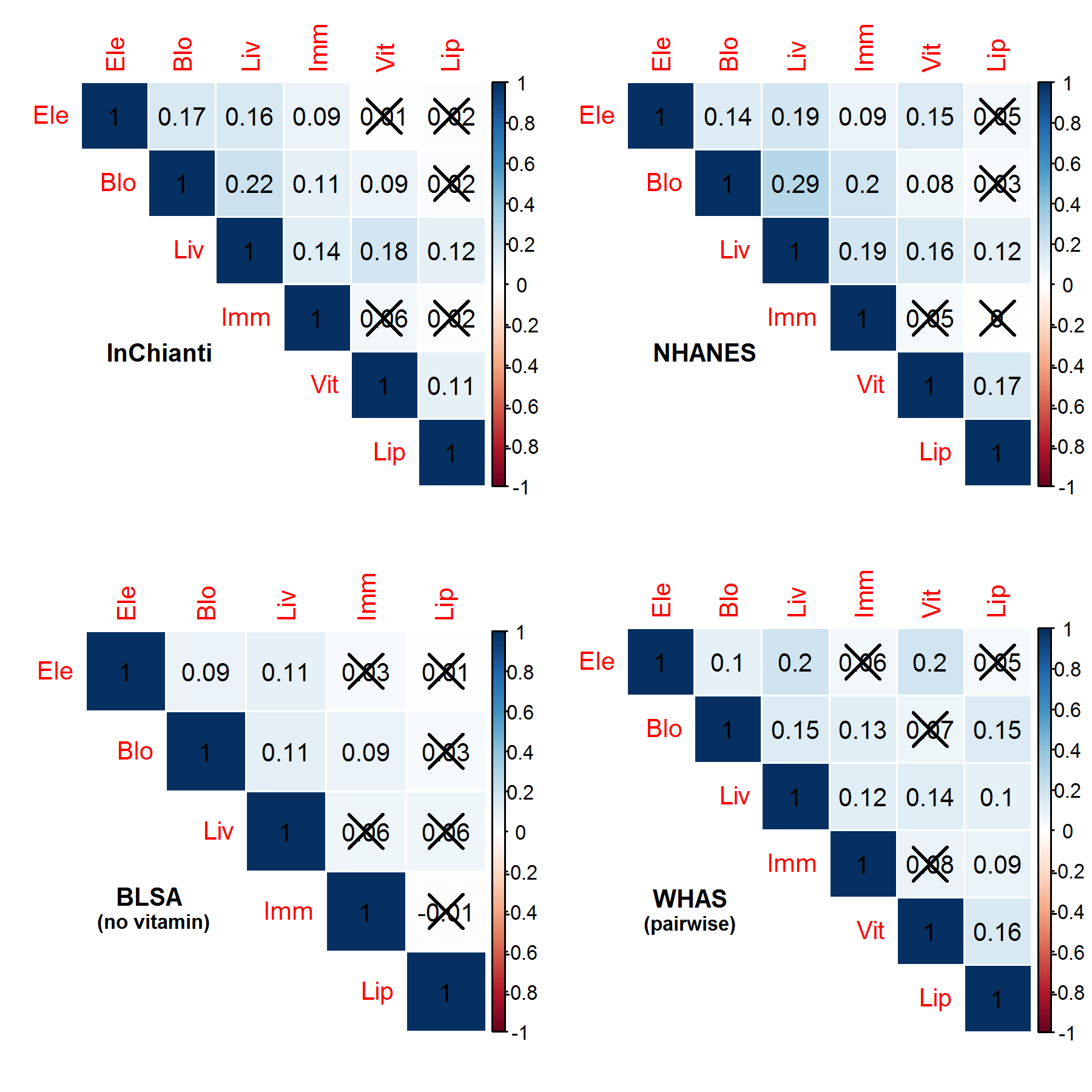

Supplement: Supplementary file 1 — Fig. S1 Correlations among dysregulation scores of the a priori systems. The only difference from Fig. 1 in the main text is we did not adjust for age. [file ACEL-14-1103-s001.tif]
