# Supplementary material for: Homeostatic dysregulation proceeds in parallel in multiple physiological systems
Source: Aging Cell. 2015 Sep 29;14(6):1103–12. doi: 10.1111/acel.12402 (PMC4693454; doi:10.1111/acel.12402)
Supplement: Supplementary file 2 — Table S1 Results from structural equations models showing lagged effects of dysregulation across systems. Appendix S1 Additional details on data sets, health outcomes measures, and structural equations models. [file ACEL-14-1103-s002.docx]

Supporting Information for

Homeostatic dysregulation proceeds in parallel in multiple physiological systems

Qing Li, Shengrui Wang, Emmanuel Milot, Patrick Bergeron, Luigi Ferrucci, Linda P. Fried and Alan A. Cohen

**Data sets**

The Baltimore Longitudinal Study of Aging (BLSA) is one of the world's best known, oldest, and longest longitudinal studies of aging in humans, continuing since 1958. Participants are community-dwelling adults in the Baltimore and Washington DC areas aged 21-96, primarily Caucasians from middle- or upper-middle-class backgrounds. Follow-up is approximately every two years. A 2003 re-design of methodology was tailored to improve the inference for systems-level questions.

The Women’s Health and Aging Study (WHAS) is a population-based prospective study of community-dwelling women. Originally, WHAS was two separate studies, WHAS I including 1002 women aged 65+ among the 1/3 most disabled in the population, and WHAS II including 436 women aged 70-79 among the 2/3 least disabled. The participants were drawn from eastern Baltimore City and Baltimore County, Maryland. Baseline assessment occurred from November 1992 to February 1995 in WHAS I and from August 1994 to February 1996 in WHAS II. Follow-ups were conducted roughly 1.5, 3, 6, 7.5, and 9 years later. For this study, WHAS I and WHAS II were combined into a single data set consisting of all visits of all individuals with sufficient biomarker data for analysis.

Invecchiare in Chianti (InCHIANTI) is a prospective population-based study of 1156 adults aged 65-102 and 299 aged 20-64 randomly selected from two towns in Tuscany, Italy using multistage stratified sampling in 1998. Follow-up blood and urine samples were taken in 2001-03, 2005-06, and 2007-08.

National Health and Nutrition Examination Survey (NHANES) is a cross-sectional study based on a representative sample of the US population and conducted in various waves since the 1970s. We used six waves: 1999-2000, 2001-2002, 2003-2004, 2005-2006, 2007-2008, and 2009-2010. For markers with consistent measurement protocols, data were combined across waves; otherwise, data from each wave were treated as separate variables.

**Health status measures**

Health status measures were not available for BLSA. Type and availability of data varied substantially between WHAS and InCHIANTI; variables were defined as follows:

***Diabetes***

For InCHIANTI, prevalent diabetes was defined as definite (score of 1) when fasting plasma glucose level was ≥126 mg/dL, based on the American Diabetes Association 2003 criteria, or when there was use of diabetes drugs (insulin and analogues, or oral hypoglycemics). A score of 0.5 (possible diagnosis) was attributed to self-reported diabetes and treatment of diabetes through diet. Diabetes diagnosis was assumed to apply to all future visits. For WHAS, diabetes positivity was based on self-reported physician diagnosis. Thus, in InCHIANTI each individual had a score or 0, 0.5, or 1 at each time point; in WHAS, each individual had a score of 0 or 1 at baseline.

***Cancer***

For InCHIANTI, the presence of cancer was defined through self-reported malignant neoplasms (ICD-9 codes 140 through 208 inclusively, except 173). We retained only recent diagnoses, i.e. within 5 years. For follow-up visits, newly diagnosed cancers that occurred within the last three years were considered. In WHAS, the presence of cancer was defined through self-reported physician diagnosis. Thus, in InCHIANTI each individual had a score or 0 or 1 at each time point; in WHAS, each individual had a score of 0 or 1 at baseline.

***Cardiovascular disease***

For InCHIANTI, cardiovascular disease (CVD) was considered positive when at least one of the following was present: angina pectoris, myocardial infarction, congestive heart failure (CHF), stroke, peripheral arterial disease (PAD) or severe stenosis on coronary angiography. The presence of angina pectoris was defined as definite (score of 1) when there was use of organic nitrates and either one of the following: self-reported diagnosis, documentation or Rose Angina Questionnaire (ROSANG) score. If only one of medication, self-report, documentation or ROSANG score was present, a possible diagnosis was given (score of 0.5). We only retained angina pectoris diagnosed within the previous year or under current treatment. The presence of myocardial infarction was considered definite when there were signs of necrosis on electrocardiography (ECG), documented and self-reported diagnosis, aorto-coronary bypass or angioplasty. A possible score was attributed when only self-report was present. Only diagnoses within the previous year were retained. A definite score for CHF was attributed when self-report and physical exam or documentation were present or when any of those three, in addition to medication (diuretics or aldosterone antagonists and angiotensine II antagonists, angiotensin-converting-enzyme inhibitors or digitalis glycosides) or evidence in the ECG exam (presence of necrosis, atrial fibrillation in the cardiac rhythm, incomplete or complete L bundle branch blk in the intraventricular conduction, or L ventricular hypertrophy or overload), was present. A possible CHF score was given when only one of the aforementioned criteria was met. Stroke diagnosis was based on self-report, physical exam and documentation; when two or more criteria were present, a definite score was given while a possible score was attributed when only one diagnostic criterion was met. Transient ischemic attack (self-report) was considered as possible stroke. A definite score for PAD was given to ankle-brachial index (lower blood pressure of the two legs) lower than 0.9. A possible score was attributed to evidence in the physical exam, documentation or Rose PAD questionnaire. The Rose PAD questionnaire score was calculated according to responses to Rose claudication items (pain in legs while walking, standing still or sitting, pain in the calf and pain in the leg or foot at night). Diagnoses for angina pectoris, CHF, stroke, PAD and severe stenosis were adjusted across follow-up visits, as described above. In WHAS, CVD was considered positive when at least one the following was present: myocardial infarction, angina pectoris, CHF, heart disease, or stroke. All diagnoses were based on self-report. Thus, in InCHIANTI each individual had a score or 0, 0.5, or 1 at each time point; in WHAS, each individual had a score of 0 or 1 at baseline.

***Frailty***

We used Fried’s frailty criteria (Fried et al., 2001) to assign a number of criteria (between 0 and 5). This measure was available at baseline in InCHIANTI and at each visit in WHAS. Fried’s frailty criteria are unintentional weight loss, fatigue, reduced grip strength, reduced physical activity, and low gait speed; fulfilling three or more of these criteria indicates clinical frailty, and fulfilling 1-2 indicates a pre-frail state. We use the number of criteria rather than frailty state in order to maximize statistical power; similar results are obtained using a dichotomous frail-non-frail outcome.

***Comorbidities***

For InCHIANTI, the number of comorbidities included definite diagnoses for the following: cancer, chronic liver disease (self-report), angina pectoris, myocardial infarction, CHF, stroke, diabetes mellitus (defined as blood glucose ≥140 mg/dL or use of diabetes drugs), chronic bronchitis or emphysema, profoundly impaired renal function (creatinine clearance assessed via Cockcroft-Gault formula ≤30 mL/min), severe stenosis, kidney failure (self-report), deep venous thrombosis (DVT) and thyroid disease. For WHAS, the number of comorbidities included any positive diagnosis for cancer, angina pectoris, myocardial infarction, CHF, stroke, diabetes mellitus, heart disease, and lung disease, all of which were based on self-reported diagnosis. Thus, for InCHIANTI each individual had a score between 0 and 13 at each time point; for WHAS, each individual had a score between 0 and 9 at baseline.

**Additional analyses**

**Unadjusted correlations among dysregulation scores**

In the main text we presented the age-adjusted correlations among dysregulation scores, in order to stress the intrinsic correlation among the systems. Here we show the figure (Supplementary Figure 1) without adjusting for age.

**Structural Equation Models**

In the main text we showed semi-independence of the systems. Another important issue is inter-temporal relations of the dysregulation scores; in other words, taking two time points t1 and t2, what is the effect of the dysregulation scores in t1 on the dysregulation scores in t2. It is natural to expect a positive effect on the same system, for example, higher dysregulation score in the electrolytes in t1 leads to higher dysregulation score in t2; however, the signs and magnitudes of the inter-temporal effects of electrolyte dysregulation score on other dysregulation scores are not clear. If the physiological systems are semi-independent, it is natural to expect some significant causal effects across different systems. A causality analysis is necessary in this case. Structural equation model is one of the most often used models in causal analysis. In our case, the effect is “one-way”, meaning that the dysregulation scores in t1 could have a causal effect on the dysregulation scores in t2 but not reversely. The analysis requires longitudinal data of two visits (at least); BLSA has very little information on vitamins and WHAS has very little information on vitamins apart from the first visit. For this reason we included only five of the six systems, i.e., we excluded the vitamin system in this analysis. With five systems, we have five regression equations; in each equation the dependent variable is the dysregulation score in t2 for a certain system and the five independent variables are the five dysregulation scores of the five systems in t1. This gives the possibility of 5*5=25 causal effects. In addition, it is likely that the five equations are not independent and so it is reasonable to allow correlations between the different equations. For this reason we take the Seemingly Unrelated Regression (SUR) of Zellner (1962), which is a special case of structural equation model (SEM). InChianti has rich information on vitamins for t1 and t2, so we also perform the analysis with six systems (equations) for InChianti in order to compare.

The results show two things, first, there is clearly a positive effect of dysregulation scores in t1 on dysregulation scores of the same system in t2. The effect is often around 0.5 and is always significant. This means a higher dysregulation score in t1 results in a higher dysregulation score in t2 for the same system. Second, there are certainly significant effects among some of the inter-system effects, the magnitude of such effects are often ten times smaller than the intra-system effects; however, the results are not concordant from one dataset to another. This is almost certainly because the five systems represent only a small fraction of underlying physiological systems and so we could not fully control for inter-system interactions. For example, when we add a sixth system (vitamins) for InChianti, the inter-system results changed: the “Liver -> Electrolyte” effect is no longer significant while the “Blood -> Liver” effect becomes significant; we see in the meantime two significant effects related to the vitamin system. For this reason we do not include the results in the main text; nevertheless the results do support the hypothesis of system-level dysregulation as well as semi-independence of the different systems, although we cannot fully describe the inter-temporal semi-independence among the systems.

**Supplementary Figure Legends**

Supplementary Figure 1: Correlations among dysregulation scores of the a priori systems. The only difference from Figure 1 in the main text is we did not adjust for age.

**Supplementary References**

Fried LP, Tangen CM, Walston J, Newman AB, Hirsch C, et al. (2001) Frailty in Older Adults: Evidence for a Phenotype Journal of gerontology Series A, Biological sciences and medical sciences 56: M146-M157.

Zellner, A. (1962), An efficient method for estimating seemingly unrelated regressions and tests for aggregate bias, Journal of the American Statistical Association 57, 348–368.

**Supplementary Table 1: Results from structural equations models showing lagged effects of dysregulation across systems**

| **System T1 → System T2** | | **BLSA (5 sys.)** | |  | **WHAS (5 sys.)** | |  | **InChianti (5 sys.)** | |  | **NHANES (6 sys.)** | |
| --- | --- | --- | --- | --- | --- | --- | --- | --- | --- | --- | --- | --- |
|  |  | **Effect** | **p - value** |  | **Effect** | **p - value** |  | **Effect** | **p - value** |  | **Effect** | **p - value** |
| Electrolytes → | |  |  |  |  |  |  |  |  |  |  |  |
|  | Electrolytes | **0.28** | **<0.0001** |  | **0.39** | **<0.0001** |  | **0.19** | **<0.0001** |  | **0.19** | **<0.0001** |
|  | Blood | **0.09** | **0.02** |  | 0.01 | 0.72 |  | 0.03 | 0.51 |  | 0.03 | 0.47 |
|  | Liver | 0.04 | 0.35 |  | **0.10** | **0.008** |  | 0.07 | 0.09 |  | 0.07 | 0.12 |
|  | Immune | 0.02 | 0.58 |  | 0.00 | 0.91 |  | 0.04 | 0.34 |  | 0.03 | 0.40 |
|  | Lipid | 0.01 | 0.74 |  | -0.01 | 0.71 |  | -0.08 | 0.06 |  | **-0.09** | **0.03** |
|  | Vitamins |  |  |  |  |  |  |  |  |  | 0.02 | 0.63 |
| Blood → | |  |  |  |  |  |  |  |  |  |  |  |
|  | Electrolytes | -0.02 | 0.48 |  | **0.06** | **0.05** |  | 0.03 | 0.35 |  | 0.03 | 0.30 |
|  | Blood | **0.57** | **<0.0001** |  | **0.46** | **<0.0001** |  | **0.61** | **<0.0001** |  | **0.61** | **<0.0001** |
|  | Liver | 0.04 | 0.25 |  | 0.04 | 0.24 |  | 0.00 | 0.88 |  | -0.01 | 0.85 |
|  | Immune | 0.02 | 0.58 |  | 0.00 | 0.93 |  | **-0.06** | **0.04** |  | **-0.07** | **0.03** |
|  | Lipid | -0.04 | 0.17 |  | **0.07** | **0.04** |  | -0.03 | 0.39 |  | -0.04 | 0.26 |
|  | Vitamins |  |  |  |  |  |  |  |  |  | **0.09** | **0.008** |
| Liver → | |  |  |  |  |  |  |  |  |  |  |  |
|  | Electrolytes | 0.03 | 0.49 |  | **0.07** | **0.03** |  | -0.04 | 0.18 |  | -0.04 | 0.25 |
|  | Blood | 0.05 | 0.25 |  | 0.02 | 0.51 |  | **0.11** | **0.001** |  | **0.11** | **0.001** |
|  | Liver | **0.47** | **<0.0001** |  | **0.59** | **<0.0001** |  | **0.57** | **<0.0001** |  | **0.57** | **<0.0001** |
|  | Immune | 0.01 | 0.74 |  | 0.04 | 0.28 |  | 0.04 | 0.15 |  | 0.05 | 0.11 |
|  | Lipid | 0.01 | 0.82 |  | 0.04 | 0.21 |  | 0.01 | 0.85 |  | 0.00 | 0.95 |
|  | Vitamins |  |  |  |  |  |  |  |  |  | 0.04 | 0.24 |
| Immune → | |  |  |  |  |  |  |  |  |  |  |  |
|  | Electrolytes | 0.01 | 0.82 |  | **0.09** | **0.02** |  | 0.00 | 0.98 |  | 0.00 | 0.94 |
|  | Blood | 0.02 | 0.66 |  | 0.04 | 0.23 |  | -0.02 | 0.62 |  | -0.02 | 0.66 |
|  | Liver | 0.00 | 0.98 |  | 0.00 | 0.99 |  | 0.02 | 0.70 |  | 0.00 | 0.95 |
|  | Immune | **0.45** | **<0.0001** |  | **0.28** | **<0.0001** |  | **0.39** | **<0.0001** |  | **0.38** | **<0.0001** |
|  | Lipid | 0.01 | 0.70 |  | -0.02 | 0.56 |  | 0.05 | 0.19 |  | 0.04 | 0.36 |
|  | Vitamins |  |  |  |  |  |  |  |  |  | **0.13** | **0.001** |
| Lipid → | |  |  |  |  |  |  |  |  |  |  |  |
|  | Electrolytes | **0.09** | **0.02** |  | -0.04 | 0.33 |  | -0.01 | 0.70 |  | -0.01 | 0.75 |
|  | Blood | 0.01 | 0.79 |  | 0.01 | 0.80 |  | 0.04 | 0.29 |  | 0.05 | 0.22 |
|  | Liver | 0.02 | 0.53 |  | 0.05 | 0.18 |  | 0.05 | 0.16 |  | 0.06 | 0.11 |
|  | Immune | 0.02 | 0.65 |  | 0.02 | 0.59 |  | -0.02 | 0.52 |  | -0.01 | 0.72 |
|  | Lipid | **0.38** | **<0.0001** |  | **0.60** | **<0.0001** |  | **0.50** | **<0.0001** |  | **0.49** | **<0.0001** |
|  | Vitamins |  |  |  |  |  |  |  |  |  | -0.05 | 0.16 |
| Vitamins → | |  |  |  |  |  |  |  |  |  |  |  |
|  | Electrolytes |  |  |  |  |  |  |  |  |  | 0.00 | 0.93 |
|  | Blood |  |  |  |  |  |  |  |  |  | 0.07 | 0.09 |
|  | Liver |  |  |  |  |  |  |  |  |  | **0.08** | **0.05** |
|  | Immune |  |  |  |  |  |  |  |  |  | -0.04 | 0.34 |
|  | Lipid |  |  |  |  |  |  |  |  |  | -0.04 | 0.30 |
|  | Vitamins |  |  |  |  |  |  |  |  |  | **0.32** | **<0.0001** |
| Coefficients and their associated p-values as estimated from structural equations models that estimate the effect of dysregulation in each system at Time 1 (T1) on dysregulaion in each system at Time 2 (T2). Note that T1 and T2 are not necessarily the first and second visit; T2 is one visit after T1. Thus in some cases T1 may represent visit 3 and T2 visit 4, for example. | | | | | | | | | | | | |
